# Chemokine CXCL8 Promotes HIV-1 Replication in Human Monocyte-Derived Macrophages and Primary Microglia *via* Nuclear Factor-κB Pathway

**DOI:** 10.1371/journal.pone.0092145

**Published:** 2014-03-24

**Authors:** Manmeet K. Mamik, Anuja Ghorpade

**Affiliations:** Department of Cell Biology and Immunology, University of North Texas Health Science Center, Fort Worth, Texas, United States of America; University of Missouri-Kansas City, United States of America

## Abstract

**Background:**

Chemokine CXCL8 is an important neutrophil chemoattractant implicated in various neurodegenerative disorders. Cytokine/chemokine imbalance, with an increase in proinflammatory cytokines like interleukin-1β and tumor necrosis factor-α within the central nervous system, is a hallmark of human immunodeficiency virus (HIV)-1 infection. We previously reported that HIV-1 infection is linked to upregulation of CXCL8 in brain tissues and human astrocytes. Chemokines play crucial roles in trafficking of leukocytes and trafficking of HIV-1-infected across the blood-brain barrier play an important role in HIV-1 central nervous system disease. In the post-antiretroviral therapy era, low level of productive replication of HIV-1 in brain is a critical component of neuropathogenesis regulation. The present study investigated the effect of CXCL8 on productive infection of HIV-1 in human monocytes-derived macrophages (MDM) and primary human microglia.

**Results:**

Human MDM and microglia were infected with the blood or brain derived HIV-1 isolates, HIV-1_ADA_ or HIV-1_JRFL_. Treatment with CXCL8 significantly upregulated HIV-1*p24* levels in supernatants of both HIV-1-infected MDM as well as microglia. In addition, the formation of 2-long terminal repeat (LTR) circles, a measure of viral genome integration, was significantly higher in CXCL8-treated, HIV-1-infected MDM and microglia. Transient transfection of U937 cells with HIV-1 LTR luciferase reporter construct resulted in increased promoter activity when treated with CXCL8. Moreover, increased nuclear translocation of nuclear factor-κB was seen in HIV-1-infected MDM following CXCL8 treatment. Blocking CXCL8 receptors CXCR1 and CXCR2 abrogated the CXCL8-mediated enhanced HIV-1 replication.

**Conclusion:**

Our results show that CXCL8 mediates productive infection of HIV-1 in MDM and microglia *via* receptors CXCR1 and CXCR2. These results demonstrate that CXCL8 exerts its downstream effects by increasing translocation of nuclear factor-κB into the nucleus, thereby promoting HIV-1 LTR activity.

## Introduction

Human immunodeficiency virus (HIV)-1 infects CD4+ T cells and monocytes in peripheral blood, which differentiate into tissue specific macrophages. Microglia, the resident macrophages of the brain, and perivascular macrophages that migrate into the brain are prime targets for HIV-1 productive infection in the brain [1], [2]. The glycoprotein (gp) 120 in the viral envelope binds to CD4 receptor on host cells. Macrophage tropic viruses primarily use CCR5 as a co-receptor [3], [4], [5]. HIV-1 replication is a complex mechanism involving both host and viral factors. In the central nervous system (CNS), astrocytes are not productively infected and the neurons are not targets for HIV-1 infection [6], [7]. Therefore, majority of viral replication in CNS occurs in perivascular macrophages and/or microglia within brain parenchyma [8], [9], [10]. Due to poor penetration of anti-retroviral drugs and other factors, macrophages and/or microglia continue to harbor and release infectious viral particles, viral proteins and other soluble factors, which are potentially neurotoxic and lead to inflammation in CNS [11], [12]. Although low plasma levels of HIV-1 are maintained by anti-retroviral therapy, intracellular reservoir of virus persists. Immune activation markers such as interleukin (IL)-6 and sCD14 determine the level of viral replication in HIV-1 infected population [13]. Persistence of HIV-1 in the brain gradually leads to HIV-associated neurocognitive disorders (HAND) in almost 50% of infected individuals [14]. Thus, complete understanding of factors contributing towards HIV-1 replication in CNS is important for better therapeutic strategies to combat HAND.

HIV-1 viral proteins affect inflammatory responses by altering cytokine and chemokine production [15], [16]. Chemokine CXCL8 is one of the first chemokines found in the brain and is produced by almost all cells in CNS; astrocytes, microglia and neurons [17], [18], [19]. Elevated levels of CXCL8 have been reported in plasma, serum and cerebrospinal fluid of HIV-1-infected individuals suggesting its potential role in neuroinflammatory processes and neurodegeneration in HAND [20], [21]. Increase in proinflammatory cytokines like IL-1β, IL-6 and tumor necrosis factor (TNF)-α follows soon after initial HIV-1 infection. A previous study from our group indicated that CXCL8 expression is robustly increased in astrocytes treated with IL-1β and TNF-α by src homology-2 domain-containing protein tyrosine phosphatase and mitogen activated protein kinases pathways [22]. In the present study, we extended this observation to unravel effect of CXCL8 on HIV-1 replication in human monocyte-derived macrophages (MDM) and primary human microglia. Cytokines and chemokines have been shown to induce HIV-1 replication in variety of cell types [23], [24]. TNF-α alters permeability of blood-brain barrier that allows infiltration of HIV-1 infected cells into the brain [25]. Stimulation of HIV-1 replication by CXCL8 has been reported in macrophages and T-lymphocytes [20]. However, the mechanisms linking enhanced chemokine levels and HIV-1 replication are not well understood. While there has been considerable development in understanding of mechanisms that regulate CXCL8 production in CNS, more detailed investigation into the role of this chemokine on HIV-1 replication is required.

Various HIV-1 isolates have been widely used to study HIV-1 infection in macrophages and microglia [26], [27]. Blood and brain derived isolates HIV-1_ADA_ and HIV-1_JRFL_ preferentially infect mononuclear phagocytes and both were selected for our present study for investigation of strain-dependent effects from a panel of macrophage-tropic viral isolates. The promonocytic cell line U937, characterized by ability to differentiate into macrophages, forms an *in vitro* model system for transfection studies, which have always been challenging to be carried out in human macrophages [28]. Previous studies have shown that chemokines like CCL3 and CCL5 suppress HIV-1 replication in T cells and monocytes [29], [30]. In contrast some reports have shown such chemokines to aid in viral replication [31], [32], [33]. We show that CXCL8 increases translocation of nuclear factor (NF)-κB into the nucleus thereby increasing HIV-1 long terminal repeat (LTR) activity. HIV-1 LTR promoter is known to have NF-κB binding sites facilitated by chromatin accessibility at the LTR promoters [34]. We also show that the CXCL8 receptors CXCR1 and CXCR2 are important for downstream events initiated by CXCL8 in macrophages. The data demonstrate that CXCL8 can potentiate HIV-1 replication in both human macrophages and microglia *via* NF-κB dependent mechanisms. The present study provides insight into the role of chemokines in HIV-1 infection in the brain and thus their contribution towards HIV-1 associated neurodegeneration.

## Methods

### Isolation and cultivation of primary human microglia

Human microglia were isolated as previously described [35], [36], [37], [38] from first- and early second-trimester aborted specimens, ranging from 14 to 20 weeks, obtained from the Birth Defects Laboratory, University of Washington, Seattle, WA in full compliance with the ethical guidelines of the NIH and with the Helsinki Declaration of 1975 (in its most recently amended version). The institutional review boards of both the Universities of Washington and North Texas Health Science Center approved the collection of human tissues for research. The Birth Defects Laboratory obtained written consent from all tissue donors. Briefly, tissue was dissected mechanically followed by several Hank's balanced salt solution washes. Tissue pieces were dissociated with 0.25% trypsin. After 14 days in culture, the nonadherent microglia cells were collected and purified by preferential adhesion. Cells were used in experimental protocols when morphological differentiation was apparent (usually 2-5 days). Microglia obtained by this procedure were routinely >98% pure as measured by immunocytochemistry staining for microglial marker CD68.

### Isolation and cultivation of human MDM

Human peripheral blood mononuclear cells (PBMC) were isolated by lymphocyte separation medium (Fisher Scientific, Waltham, MA) density centrifugation from whole blood donated by healthy volunteers (Carter Blood Care, Fort Worth, TX). Monocytes were enriched from freshly isolated PBMC using magnetic-activated cell sorting CD14+ beads and LS Columns (Miltenyi Biotec, Auburn, CA), yielding an average 98% purity. To differentiate PBMC into MDM, PBMC were cultured in Dulbecco's modified Eagle's medium (Life Technologies, Carlsbad, CA) with 10% heat-inactivated pooled human serum (Atlas Biologicals, Fort Collins, CO), 50 μg/ml gentamicin (Life Technologies), 10 μg/ml ciprofloxacin (Sigma, St. Louis, MO) and 50 ng/ml macrophage-colony stimulating factor for two weeks. MDM were cultured as adherent cells in 48-well plates (4×10^5^/well) or 6-well plates (3×10^6^) for virus infection or as non-adherent cultures in teflon flasks (2×10^6^ cells/ml, 150×10^6^ cells in flask) for transfection assays. Cultures were maintained by half-media exchange twice weekly.

### Cell lines and culture conditions

The promonocytic cell line U937 were obtained from the American Type Culture Collection (Manassas, VA) and maintained in suspension culture in RPMI-1640 (Life Technologies) supplemented with 10% (v/v) heat-inactivated fetal bovine serum and 5 μg/ml penicillin/5 μg/ml streptomycin/10 μg/ml neomycin at 37°C in a humidified atmosphere of 5% CO_2_. U937 cells were induced to differentiate by treating the cells with 20 ng/ml of phorbol 12-myristate 13-acetate (PMA) (Fisher Scientific) overnight followed by complete media exchange. Cultures were maintained by half-media exchange every 3–4 days.

### HIV-1 infection of MDM

HIV-1_JRFL_ [39] was obtained from the AIDS Research and Reference Reagent Program, Division of National Institute of Allergy and Infectious Diseases (NIAID). It was previously isolated from brain tissue of an HIV-1-infected individual with encephalitis. HIV-1_ADA_ was isolated from PBMC of an individual with AIDS and was propagated as previously described [40]. All isolates were prepared as viral stocks free of mycoplasma and endotoxin contamination. Following 2 weeks in culture, MDM were infected with HIV-1 (MOI 0.1) as described [41], [42], [43]. Following day, virus was washed off the cells and supplemented with media with or without varying concentrations (10–100 ng/ml) of human recombinant CXCL8 (R&D Systems, Minneapolis, MN). Heat-inactivated CXCL8, generated by heating at 70°C for 10 minutes, was used as a control. Heating disrupts the protein structure and thus interferes with efficient binding to its receptors. Culture supernatant samples of infected MDM were collected by half-media exchange twice weekly. Lipopolysaccharide (LPS) derived from *E.coli* (2 ng/ml, Sigma, St. Louis, MO) was used as a positive control for activation.

### DNA isolation and quantification of 2-LTR circles

To quantify 2-LTR circles, 3×10^6^ MDM were plated in 6-well plates and infected with HIV-1_ADA_ or HIV-1_JRFL_. To isolate DNA, cells were washed with phosphate-buffered saline (PBS), and DNA was harvested using the DNeasy tissue kit (Qiagen, Germantown, MD). Two-LTR circles were determined by quantitative real-time polymerase chain reaction RT-PCR using 100 ng of the DNA template and primer sets as described in [44]. The PCR products were detected with TaqMan gene expression assays using Step One Plus (Life Technologies). Absolute copy numbers were determined by normalization to standard curves generated from a serially diluted pTA2LTR/CCR5 plasmid. The plasmid pTA2LTR/CCR5 harbors the sequence of 2-LTR junction and was a generous gift from Dr. Mario Stevenson, University of Miami [45], [46]. In all cases the DNA standards were diluted into 100 ng of uninfected cellular DNA to match the cellular DNA samples.

### Plasmids and DNA transfection into MDM and U937 cells

The plasmid construct pBlue3′LTR-luc was obtained from AIDS Research and Reference Reagent Program (National Institute of Allergy and Infectious Diseases) deposited by Dr. Reink Jeeninga and Dr. Ben Berkhout [47], [48]. MDM were transfected with pBlue3′LTR-luc, *Renilla* luciferase vector (pRL, Promega), small interfering (si)RNA specific to NF-κB p65 (si-p65, Cell Signaling Technology, Danvers, MA) or scrambled non-targeting siRNA (si-Con, Thermo Scientific, Waltham, MA) using the Amaxa Human Macrophage Nucleofector kit (Lonza, Walkersville, MD). Briefly, 1 million MDM were suspended in 100 μl Nucleofector solution per cuvette with 2 μg plasmid DNA or 100 nM siRNA and transfected using a Nucleofector (Lonza) device. U937 cells were transfected using Amaxa Cell Line Nucleofector Kit C with 2 million cells/cuvette. Transfected cells were supplemented with MDM or U937 media (with 20 nM PMA) and plated in 48 well plates at a density of 4×10^5^/well. Twenty-four hours post-transfection cells were treated with indicated concentrations of CXCL8, anti-CXCL8 IgG or mouse IgG (R&D Systems).

### Measurement of HIV-1 LTR promoter activity by luciferase assay

Twenty-four hours post-treatment, 4×10^5^ transfected cells/well were harvested and lysed using 65 μl lysis buffer. An aliquot (20 μl) of the cell lysate was used to measure luciferase activity using the dual-luciferase reporter assay kit (Promega, Madison, WI). The firefly luciferase activity was normalized to the *Renilla* luciferase activity. All experiments were carried out in triplicate, and the data are presented as the mean luciferase activities ± standard error of the mean (SEM).

### Quantification of CXCL8 and HIV-1p24 by ELISA

CXCL8 protein levels were determined from culture supernatants by a CXCL8 specific sandwich enzyme-linked immunosorbent assay (ELISA) (R&D Systems) and HIV-1*p24* ELISA (Advanced Biosciences Laboratories, Rockville, MD) according to manufacturer's protocol. Absorbance was determined by Spectromax M5 microplate reader using SoftMax Pro V5 software (Molecular Devices, Sunnyvale, CA).

### Western blot analysis

MDM were cultured as adherent monolayers in 6 well-plates at a density of 3×10^6^ cells/well and infected with HIV-1_ADA_ overnight. Following day, cells were washed with PBS and supplemented with fresh medium with or without CXCL8. LPS (2 ng/ml) was added to uninfected MDM. Three hours later, cells were collected by scraping in PBS. Cytoplasmic and nuclear protein extracts were isolated using nuclear and cytoplasmic extraction kit (Thermo Fisher Scientific, Pittsburgh, PA). Equal amounts of protein samples (15 μg) were boiled with 4× NuPAGE loading sample buffer (Life Technologies) for 5–10 min, resolved by NuPage 4–12% Bis tris gel and subsequently transferred to a nitrocellulose membrane using i-Blot (Life Technologies). The membrane was incubated with primary antibody against NF-κB p65 (1∶1000, Cell Signaling) overnight at 4°C, washed and then incubated with anti-mouse or anti-rabbit goat antibody IgG conjugated to horseradish peroxidase (1∶10,000, Bio-Rad) for 2 h at room temperature. The membrane was then developed with SuperSignal west femto substrate (Fisher Scientific) in a Fluorochem HD2 Imager (ProteinSimple, Inc. Santa Clara, CA). GAPDH (1∶1000, Santa Cruz Biotechonology) and Lamin A/C (1∶1000, Cell Signaling Technology) were used as loading controls for cytoplasmic and nuclear extracts, respectively.

### Determination of metabolic activity

Following experimental manipulations, five percent MTT reagent (Fisher Scientific) in fresh medium was added to MDM and/or microglia and incubated for 20–45 min at 37°C. MTT is metabolically reduced to purple formazan crystals by living cells. Representative images were taken using a phase-contrast microscope. The MTT solution was removed and crystals were dissolved in dimethyl sulfoxide (Fisher Scientific) for 15 min with gentle agitation. Absorbance was then measured at 490 nm, as previously described [49].

### Immunocytochemistry

MDM and microglia were fixed with cold acetone:methanol 1∶1 (v/v) solution for 20 min at −20°C and blocked with PBS with 2% bovine serum albumin and 0.1% triton X-100 for 1 h at room temperature. Cells were then incubated with primary antibodies specific to CD68 (1∶50, Abcam, Cambridge, MA) and HIV-1*p24* (1∶50, Abcam) in blocking buffer overnight at 4°C and washed incubated with AlexaFluor secondary antibodies, anti-rabbit (488 nm, green) and anti-mouse (594 nm, red) (1∶100, Life Technologies). Nuclei were visualized with diamidino-2-phenylindole (DAPI; 1∶800, Life Technologies). Micrographs were taken on a Nikon Eclipse Ti-4 (Nikon Inc., Melville, NY) using the NLS-Elements BR. 3.0 software.

### Statistical analyses

Statistical analyses were carried out using Prism V5.0 (GraphPad Software, La Jolla, CA) with one-way analysis of variance (ANOVA) and Newman-Keuls post-test for multiple comparisons. Significance was set at p<0.05 and data represents means +/− SEM. Data presented is representative of a minimum of three independent experiments with two or more independent donors.

## Results

### Chemokine CXCL8 increases HIV-1p24 levels in MDM

**Figure 1:**
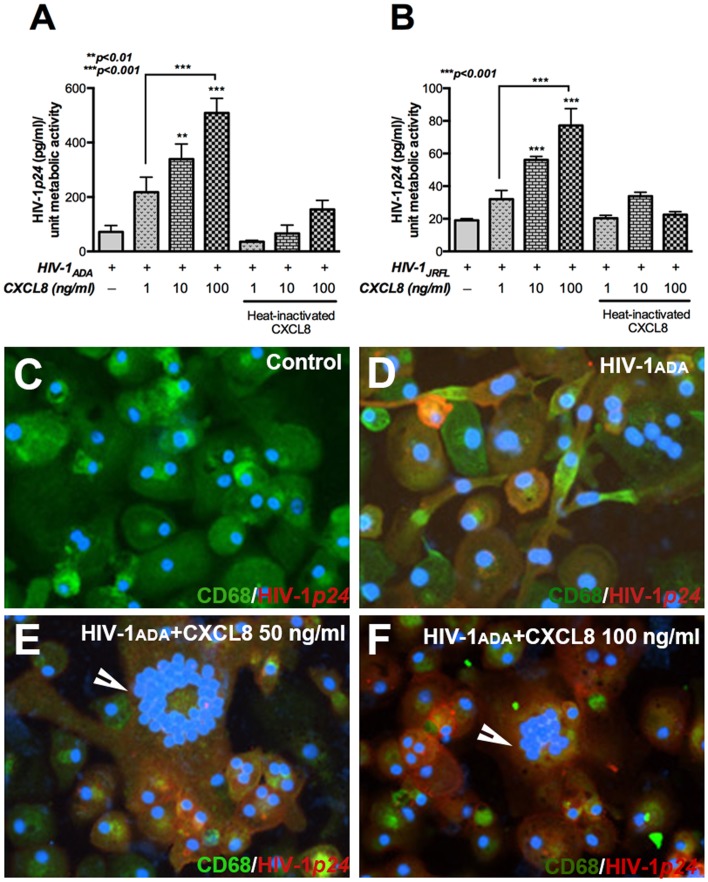
CXCL8 induces a dose-dependent upregulation of HIV-1*p24* in HIV-1-infected MDM. Human MDM were infected with HIV-1_ADA_ or HIV-1_JRFL_ (MOI 0.1) overnight, followed by treatment with indicated concentrations of CXCL8. Cell culture supernatants were collected 1 week post-infection and HIV-1*p24* levels were measured by ELISA in MDM infected with (**A**) HIV-1_ADA_ or (**B**) HIV-1_JRFL_. Expression of macrophage marker CD68 and HIV-1*p24* was measured by immunocytochemistry in HIV-1_ADA_-infected MDM 1 week post-infection. Nuclei were stained in blue by DAPI. (**C**) Control, CD68-positive cells (green) (**D**) Co-localization (yellow) of CD68 and HIV-1*p24* (red) in HIV-1_ADA_-infected MDM (**E, F**) HIV-1*p24* expression and multinucleated giant cells (represented by arrowhead) in CXCL8-treated, HIV-1_ADA_-infected MDM. Original magnification 200X. Results are expressed as mean ± SEM, analyzed by one-way ANOVA and Newman-Keuls post-test for multiple comparisons.

Secretion of viral antigen HIV-1*p24* is an important indicator of viral protein production by infected cells. We examined HIV-1*p24* levels in MDM infected with macrophage tropic blood-derived isolate HIV-1_ADA_ and brain-derived isolate HIV-1_JRFL_. To determine the role of CXCL8 in HIV-1 infection, MDM were infected with HIV-1_ADA_ or HIV-1_JRFL_ followed by treatment with or without varying concentrations of CXCL8. HIV-1*p24* levels were measured 1 week post-infection by ELISA. As shown in Figure 1A and B, MDM treated with CXCL8 showed significant dose-dependent increase in HIV-1*p24* levels when compared to infected cells without CXCL8 treatment. Significant increase in HIV-1*p24* was observed with CXCL8 concentration as low as 10 ng/ml, for both HIV-1_ADA_ and HIV-1_JRFL_ infection (p<0.01 and 0.001 respectively). HIV-1*p24* levels were increased by more than 2-fold when CXCL8 concentration was increased from 1 to 100 ng/ml for both HIV-1_ADA_- and HIV-1_JRFL_-infected MDM (p<0.001). Also, the basal levels of HIV-1*p24* were more in HIV-1_ADA_-infected MDM as compared to HIV-1_JRFL_-infected MDM, which is in agreement with previous findings demonstrating higher infectivity with HIV-1_ADA_ as compared to HIV-1_JRFL_ [50]. Heat-inactivated CXCL8, used as a control, did not significantly alter HIV-1*p24* levels in infected MDM. In parallel, we investigated the cellular expression of HIV-1*p24* in CXCL8-treated HIV-1-infected MDM. One week post-infection, MDM were immunoassayed for HIV-1*p24* (red) and macrophage marker CD68 (green). *HIV-1p24* antigen expression could be seen in HIV-1-infected MDM but as expected, not in control cells (Figure 1C, D). Furthermore, CXCL8-treated MDM showed brighter HIV-1*p24* staining and multinucleated giant cells (MGC) as compared to infected MDM without CXCL8 treatment (Figure 1E, F). Taken together, CXCL8 treatment led to dose-dependent increase in HIV-1*p24* release as well as enhanced cellular expression in HIV-1 infected MDM.

### Endogenous CXCL8 stimulates HIV-1p24 levels in HIV-1-infected MDM

**Figure 2:**
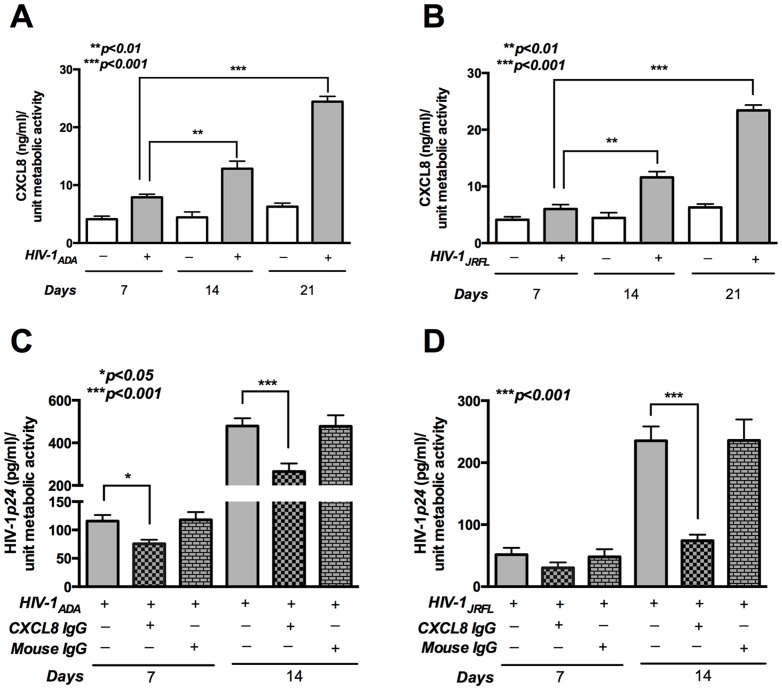
Endogenous CXCL8 stimulates HIV-1*p24* levels in HIV-1-infected MDM. Human MDM were infected with HIV-1_ADA_ or HIV-1_JRFL_ (MOI 0.1) overnight, followed by treatment with/without anti-CXCL8 IgG (1.2 μg/ml). Mouse IgG (1.2 μg/ml) was used as a control. Cell culture supernatants were collected at indicated time points. CXCL8 levels were determined by ELISA in MDM infected with (**A**) HIV-1_ADA_ or (**B**) HIV-1_JRFL_ (**C**) HIV-1*p24* levels as determined by ELISA in MDM infected with HIV-1_ADA_ or (**D**) HIV-1_JRFL_. Results are expressed as mean ± SEM, analyzed by one-way ANOVA and Newman-Keuls post-test for multiple comparisons.

Chemokine CXCL8 is produced by a variety of cells including astrocytes, macrophages and microglia [17], [18], [19]. To determine whether HIV-1-infected MDM secreted CXCL8, we first measured levels in culture supernatants following HIV-1_ADA_ or HIV-1_JRFL_ infection at different time points. While the basal levels of CXCL8 in uninfected cells remained unchanged over a period of three weeks, CXCL8 significantly increased as viral infection progressed from one week to three weeks in both HIV-1_ADA_ - and HIV-1_JRFL_-infected MDM (Figure 2 A, B; p<0.001). To determine the effect of endogenous CXCL8 on HIV-1*p24* production, HIV-1-infected MDM were treated with CXCL8 neutralizing antibody the day following infection with HIV-1. Consistent with our hypothesis, by two weeks, significant downregulation in HIV-1*p24* levels was noted upon CXCL8 neutralization in both HIV-1_ADA_- and HIV-1_JRFL_-infected MDM. Treatment with mouse IgG, used as a control, did not alter HIV-1*p24* levels in both HIV-1_ADA_- and HIV-1_JRFL_-infected MDM (Figure 2 C, D; p<0.001). These results corroborated those obtained above and showed that both endogenous and exogenous CXCL8 increased HIV-1*p24* production in HIV-1-infected MDM.

### CXCL8 increases HIV-1 LTR promoter activity in U937 cells and MDM

**Figure 3:**
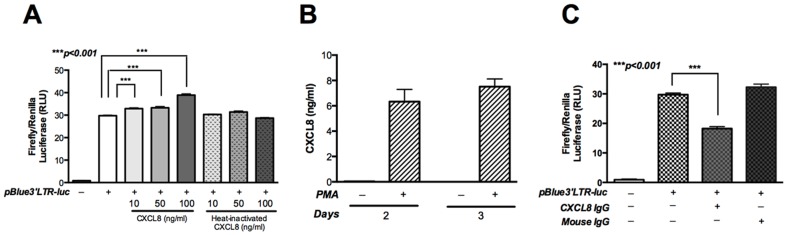
CXCL8 stimulates HIV-LTR promoter activity in U937 cells. U937 cells were transfected with pBlue3′LTR luc along with a *Renilla* transfection control. Cells were plated in 48-well plates with 20 nM PMA overnight. Following day, cells were washed and treated with indicated concentrations of CXCL8 or anti-CXCL8 IgG (1.2 μg/ml). Mouse IgG (1.2 μg/ml) was used as a control. (**A, C**) Cell lysates were collected 24 h post-treatment and relative luciferase activity was determined. (**B**) Levels of CXCL8 were measured by ELISA in PMA-treated U937 cells. Results are expressed as mean ± SEM, analyzed by one-way ANOVA and Newman-Keuls post-test for multiple comparisons.

The HIV-1 LTR promoter activity is dependent upon host cell transcriptional machinery once the viral genome integrates into the host chromosome [51], [52]. Since transcription is a requisite for production of viral proteins, we analyzed the effect of CXCL8 on HIV-1 LTR promoter activity. We utilized luciferase reporter assay with the pBlue3′LTR-luc plasmid [47], [48]. We employed the promonocytic cell line U937 for the promoter-reporter studies because of better transfection efficiency and viability following transfection. Briefly, U937 cells were cotransfected with pBlue3′LTR-luc and *Renilla* plasmid followed by treatment with varying concentrations of CXCL8 24h post-transfection. CXCL8 induced a significant dose-dependent increase in HIV-1 LTR promoter activity, as indicated by increase in luciferase activity (Figure 3A; p<0.001). No significant changes in HIV-1 LTR promoter activity were observed with heat-inactivated CXCL8. Since we observed robust increase in CXCL8 production by PMA-treated differentiating U937 cells (Figure 3B), we next evaluated the HIV-1 LTR promoter activity following neutralization of endogenous CXCL8. The luciferase activity significantly dropped upon CXCL8 neutralization (p<0.001), whereas no change was observed when control IgG was added to the transfected U937 cells (Figure 3C). In conclusion, these findings indicate an important role of chemokine CXCL8 in regulating HIV-1 LTR promoter activity.

### CXCL8 promotes formation of 2-LTR circles in HIV-1 infected MDM

**Figure 4:**
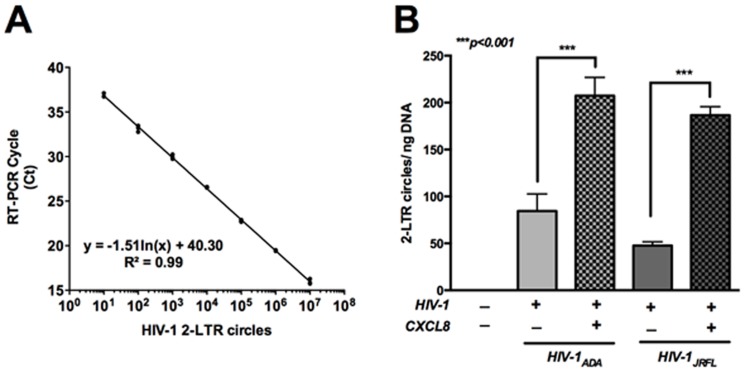
CXCL8 increases 2-LTR circle formation in HIV-1-infected MDM. Human MDM were plated in 6-well plates and infected with HIV-1_ADA_ or HIV-1_JRFL_ (MOI 0.1) overnight. Following day, MDM were treated with/without CXCL8 (50 ng/ml). DNA was extracted 1 week post-infection and 2-LTR circle junctions were amplified by real-time PCR. (**A**) Standard curve generated using pTA2LTR/CCR5 plasmid with 2-LTR copy numbers at X-axis and real-time PCR threshold counts at Y-axis. (**B**) Comparison of 2-LTR circle copy numbers in HIV-1_ADA_- or HIV-1_JRFL_-infected MDM. Results are expressed as mean ± SEM, analyzed by one-way ANOVA and Newman-Keuls post-test for multiple comparisons.

HIV-1 entry into the cells is followed by reverse transcription of viral RNA into a linear double strand cDNA copy, which is then transported into the nucleus [53], [54]. Soon after nuclear import, viral cDNA integrates into the host genome. The HIV cDNA that does not integrate then circularizes to form 2-LTR circles and can be used as a marker for nuclear import of viral DNA [55]. To investigate the effect of CXCL8 on formation of 2-LTR circles, MDM were infected with HIV-1_ADA_ or HIV-1_JRFL_ with or without CXCL8 treatment [55]. Two weeks post-infection, 2-LTR circle copy numbers were quantified by real-time PCR assay using a standard curve generated by pTA-2LTR/CCR5 (Figure 4A). We observed that treatment with CXCL8 led to significant increase in number of 2-LTR circles compared to non-treated HIV-1_ADA_- and HIV-1_JRFL_-infected MDM (Figure 4B; p<0.01). These data demonstrate that CXCL8 treatment enhanced formation of 2-LTR circles in HIV-1-infected cells along with elevated infection as evident from high HIV-1*p24* levels.

### CXCL8 treatment increased HIV-1p24 and 2-LTR circle formation in human microglia

**Figure 5:**
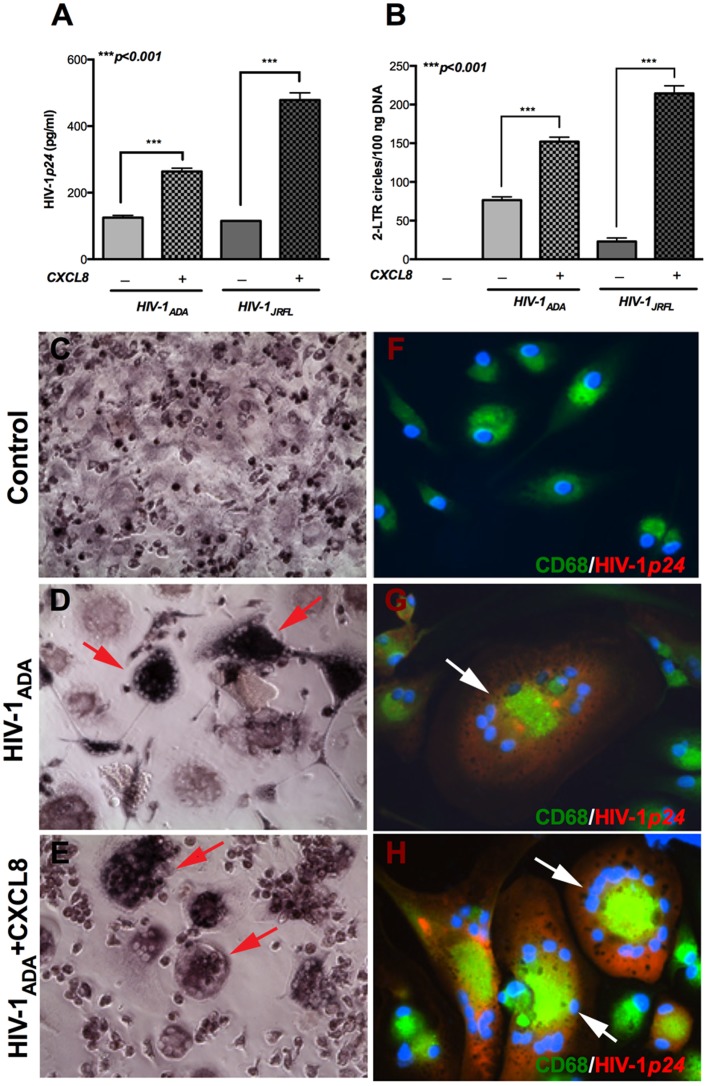
CXCL8 increases HIV-1*p24* and 2-LTR circles in HIV-1-infected primary human microglia. Primary human microglia were infected with HIV-1_ADA_ or HIV-1_JRFL_ (MOI 0.1) overnight, followed by treatment with 50 ng/ml CXCL8. (**A**) Supernatants were collected 2 weeks post-infection and HIV-1*p24* levels were measured by ELISA. (**B**) DNA was isolated and 2-LTR circle junctions were amplified by real-time PCR. (**C–E**) MTT (3-(4,5-dimethylthiazol-2-yl)-2,5-diphenyltetrazolium bromide) reagent was added to microglia and incubated for 15 minutes at 37°C. Results are expressed as mean ± SEM, analyzed by one-way ANOVA and Newman-Keuls post-test for multiple comparisons. Phase contrast pictures showing (**C**) cell morphology of uninfected primary human microglia. (**D**) Multinucleated giant cells formed with HIV-1_ADA_ infection (**E**) HIV-1_ADA_ infection and CXCL8 treatment. (**F-H**) Expression of CD68 (green) and HIV-1*p24* (red) was measured by immunocytochemistry 2 weeks post-infection with HIV-1_ADA_. Nuclei were stained in blue by DAPI. (**F**) Uninfected primary human microglia (**G**) co-localization (yellow) of CD68 and HIV-1*p24* in HIV-1_ADA_-infected microglia. (**H**) HIV-1*p24* expression and multinucleated giant cells in HIV-1_ADA_-infected microglia. Arrows represent multinucleated giant cells. Original magnification 200X.

Microglia are resident macrophages of the brain and constitute important targets for HIV-1 infection in the CNS. To confirm the results obtained in MDM and to assess whether CXCL8 promotes HIV-1 infection in human microglia, we infected cultured human microglia with viral isolates HIV-1_ADA_ or HIV-1_JRFL_. HIV-1*p24* levels, as measured by ELISA, showed significant upregulation when infected microglia were treated with CXCL8. Interestingly, CXCL8 treatment led to 2-fold increase in HIV-1*p24* levels in HIV-1_ADA_-infected microglia, whereas the increase was more than 4-fold in HIV-1_JRFL_-infected microglia (Figure 5A; p<0.001). Given the limitation in culturing microglia, only selected conditions and time-points were analyzed. Two weeks post-infection DNA was isolated and assayed for 2-LTR circles. Consistent with trends observed in HIV-1*p24* levels, CXCL8 treatment increased 2-LTR circle copy numbers by only 2-fold in HIV-1_ADA_-infected microglia as compared to a 10-fold increase in HIV-1_JRFL_-infected human microglia (Figure 5B; p<0.001). Therefore, these data corroborate the results obtained with MDM and indicate that CXCL8 treatment leads to elevated HIV-1*p24* levels and 2-LTR circles in primary human microglia.

MTT (3-(4,5-dimethylthiazol-2-yl)-2,5-diphenyltetrazolium bromide) is reduced to purple formazan by viable cells, thus formazan staining can be used to evaluate changes in cell morphology or proliferation [56]. Primary human microglia were incubated with MTT and images were taken by light microscopy. Uninfected control cells exhibited amoeboid bipolar morphology with smaller individual cells. Formazan aggregates could be seen in HIV-1_ADA_-infected microglia revealing MGC formation. Treatment with CXCL8 exacerbated formation of giant cells, as indicated by formazan aggregates (Figure 5C–E). In parallel, microglia were fixed and immunostained for CD68 and HIV-1*p24*. Intense HIV-1*p24* antigen expression could be seen in HIV-1_ADA_-infected microglia but not in control cells (Figure 5F, G). Furthermore, CXCL8-treated, HIV-1_ADA_-infected microglia showed bright HIV-1*p24* staining and MGC as compared to infected untreated cells (Figure 5H). These data demonstrate that CXCL8 enhanced HIV-1 infection in primary human microglia with both increased nuclear import of viral DNA as well as viral protein HIV-1*p24* expression.

### CXCL8 promotes HIV-1 LTR promoter activity via NF-κB dependent pathway in U937 cells and MDM

**Figure 6:**
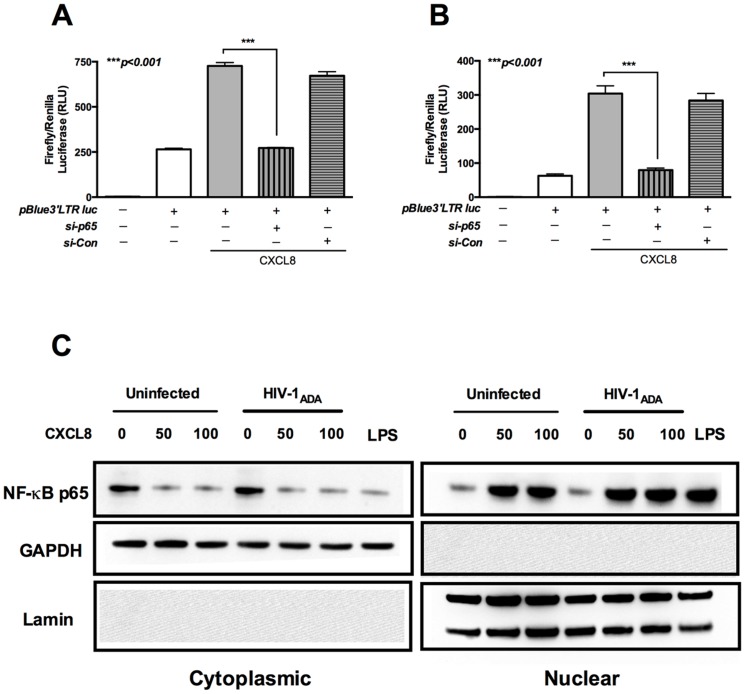
p65 subunit of NF-κB is essential for HIV-LTR promoter activity and CXCL8 translocates NF-κBp65 to the nucleus. Human MDM were transfected with pBlue3′LTR luc and siRNA for NF-κB p65. Cells were plated in 48-well plates overnight. Following day, cells were washed and treated with 50 ng/ml CXCL8. Cell lysates were collected 24 h post-treatment and relative luciferase activity was determined in (**A**) U937 cells and (**B**) Human MDM. Results are expressed as mean ± SEM, analyzed by one-way ANOVA and Newman-Keuls post-test for multiple comparisons. (**C**) HIV-1_ADA_-infected and uninfected MDM were stimulated with indicated concentrations of CXCL8 and protein lysates were collected for cytoplasmic and nuclear components 3 h post-simulation. NF-κB p65 was detected by western blotting. GAPDH and lamin were used as loading control for cytoplasmic and nuclear protein, respectively. Data are representative of two independent donors.

The HIV-1 LTR promoter controls transcription of viral genes and is reported to have NF-κB binding sites [57], [58]. The transcription factor NF-κB can translocate to nucleus in response to various internal or external stimuli [59]. The p65 subunit of NF-κB is essential in transcriptional regulation and forms a vital component of the transcription factor. We next examined the effect of silencing the p65 subunit in two cellular systems, U937 and MDM and then assayed the HIV-1 LTR promoter activity. For this, U937 cells and MDM were cotransfected with pBlue3′LTR-luc plasmid, *Renilla* and siRNA targeting p65. We found a significant reduction in CXCL8-induced HIV-1 LTR promoter activity after silencing p65 subunit of NF-κB. The trend was similar in both U937 cells and MDM (Figure 6A, B respectively; p<0.001). To further elucidate whether CXCL8 stimulated nuclear translocation of NF-κB, HIV-1_ADA_-infected and uninfected MDM were stimulated with CXCL8 and cytoplasmic and nuclear protein lysates were collected. Immunoblot analysis showed that CXCL8 served as a stimulus for the nuclear translocation of NF-κB p65 into the nucleus, and that CXCL8 facilitated this translocation in infected as well as uninfected MDMs. Together, these data demonstrate that NF-κB p65 is important for CXCL8-induced activation of HIV-1 LTR.

### CXCL8-induced enhanced HIV-1 infection is both CXCR1 and CXCR2 dependent

**Figure 7:**
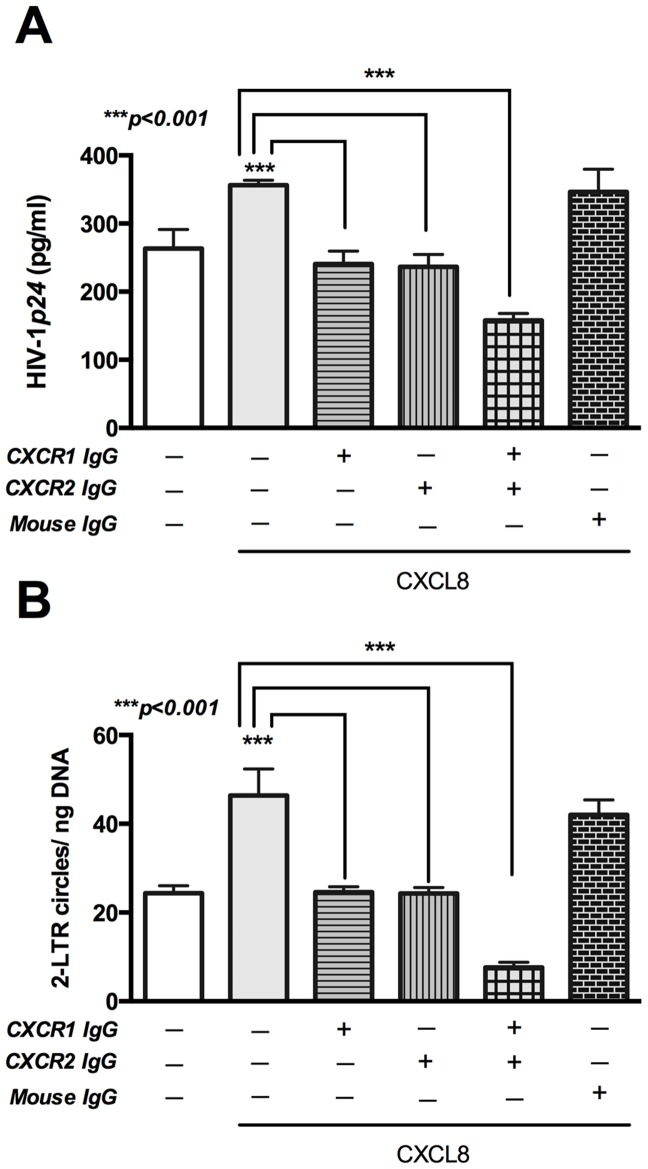
Neutralization of CXCR1 and CXCR2 reduces CXCL8-induced HIV-1*p24* levels and 2-LTR circle formation in HIV-1-infected MDM. Human MDM were infected with HIV-1_ADA_ (MOI 0.1) overnight. Following day, cells were washed and supplemented with macrophage media with/without anti-CXCR1 (20 μg/ml), anti-CXCR2 (10 μg/ml) or both and 50 ng/ml CXCL8. Mouse IgG (25 μg/ml) was used as a control. (A) Cell culture supernatants were collected 1 week post-infection and HIV-1*p24* levels were determined by ELISA. (B) DNA was extracted 1 week post-infection and 2-LTR circle junctions were amplified by real-time PCR. Results are expressed as mean ± SEM, analyzed by one-way ANOVA and Newman-Keuls post-test for multiple comparisons.

Human MDM express G-protein coupled receptors CXCR1 and CXCR2, through which CXCL8 exerts its effects [18], [60]. To evaluate the role of these receptors in CXCL8-mediated enhanced HIV-1 replication, we infected human MDM with HIV-1_ADA_ followed by addition of antibodies to CXCR1 and CXCR2. Both CXCR1 and CXCR2 played important role in CXCL8-mediated HIV-1*p24* elevation, as evident by significantly reduced HIV-1*p24* levels following CXCR1 and CXCR2 neutralization in HIV_ADA_-infected MDM (Figure 7A; p<0.001). Quantitative real-time polymerase chain reaction assay revealed similar trends in 2-LTR circle copy numbers. Neutralization of CXCR1 or CXCR2 abrogated 2-LTR circle responses, which were further reduced when both the receptors were neutralized concurrently (Figure 7B; p<0.001). These results suggest that either of the two receptors, CXCR1 and CXCR2, is required for CXCL8-mediated increase in HIV-1 infection in MDM.

## Discussion

Increased expression of chemokines has been correlated with neurodegeneration in the brain [61]. More importantly, chemokine CXCL8 has been implicated in NeuroAIDS [62]. The correlation of CXCL8 and HIV-1 infection was demonstrated very early when increased circulating levels of this chemokine were found in HIV-1-infected individuals [63], [64] and also in HIV-1 encephalitis [65]. *In vitro* studies confirmed that CXCL8 could be independently produced by HIV-1-infected microglia and astrocytes [66], [67]. HIV-1 predominantly resides and productively replicates in peripheral blood mononuclear cells and microglia, which are the resident macrophages in the brain. The infected cells release viral particles, viral proteins, cytokines and chemokines and immune response generated by them contribute towards HIV-1-associated neurodegeneration [59], [68], [69]. In the current study we investigated the mechanism of HIV-1 replication in human macrophages and microglia. We provide several lines of evidence to support the observation that CXCL8 treatment increased HIV-1 replication in human macrophages and microglia. We found that CXCL8 treatment enhanced HIV-1 LTR promoter activity in monocytic cell line U937 and MDM. The expression and secretion of structural protein HIV-1*p24* was greater with CXCL8 treatment. CXCL8 also activated transcription factor NF-κB translocation to the nucleus, which is a regulator of HIV-1 LTR promoter. We also demonstrate that both CXCR1 and CXCR2 receptors mediate CXCL8 signaling in MDM. Overall, our study shows that CXCL8 enhances HIV-1 infection in macrophages and microglia through receptors CXCR1 and CXCR2 by downstream activation of NF-κB.

We found significant effect of CXCL8 treatment on HIV-1 protein expression in infected macrophages and microglia. High levels of intracellular and secreted HIV-1*p24* suggest that CXCL8 increases viral protein expression, which is an indicator of increased viral replication [55]. The chosen CXCL8 concentrations were based on previous reports by our group and others showing robust increase in CXCL8 levels by activated astrocytes [22], [70]. Astrocytes are the major cell type of CNS contributing to elevated CXCL8 levels in brain microenvironment. Presence of CXCL8 in abundance possibly aids in migration of monocytes through blood-brain barrier leading to increased viral entry. Previous reports suggest that it may activate monocytes/macrophages and thus indirectly promote higher level of viral replication [71]. We also observed HIV-1*p24* positive MGC with CXCL8 treatment. Infected macrophages/microglia forming MGC through HIV envelope-dependent fusion are a hallmark of progressive viral infection. HIV-1*p24* positive MGC have been reported during *in vivo* expression analysis of proinflammatory cytokines in HIV-1 encephalitis brains [72]. We report increased MGC with CXCL8 treatment in HIV-1-infected macrophages and primary microglia, which suggest a plausible mechanism by which CXCL8 may contribute towards neuropathogenesis in tissue microenvironment.

The HIV-1 LTR promoter contains DNA elements, which control viral gene expression and promoter activation increases production of viral proteins and particles in the brain. We report that CXCL8 increases HIV-1 LTR promoter activity in two separate model systems, U937 cells and MDM. Monocytic cell line U937 differentiates into macrophage like cells after stimulation with agents like PMA and is an efficient model system for transfection studies. Luciferase gene under the control of HIV-1 LTR promoter showed increased activity after CXCL8 stimulation and blocking the chemokine reduced the activity. Consistent results were observed in macrophages. Viral proteins like Tat and gp120 play an important role in HIV-1 associated neurotoxicity [73], [74]. Therefore, it is plausible that CXCL8 may potentiate neurotoxicity indirectly *via* activation of HIV-1 LTR promoter, thus increasing viral protein production. HIV-1-infected macrophages/microglia themselves are a significant source of CXCL8; however, not as robust as activated astrocytes. Therefore, the observed increase in HIV-1*p24* levels may in part be contributed by endogenous CXCL8 secreted by macrophages during the course of HIV-1 infection. To address this issue, we neutralized CXCL8 in culture supernatants of HIV-1-infected macrophages and U937 cells and found decreased HIV-1*p24* levels and HIV-1 LTR promoter activity, respectively. This observation was consistent with previous report in macrophages and T-lymphocytes [20].

2-LTR circles are formed after joining of the 5′ and 3′ ends of unintegrated viral DNA. These circles are shown to be unstable in cells and serve as a useful marker for ongoing infection and viral replication in patients undergoing highly active antiretroviral therapy (ART) [45], [46]. Despite the controversy regarding stability of 2-LTR circles, the level of 2-LTR circles is proportional to the level of nuclear import of viral DNA [75]. The observed rise in 2-LTR circles possibly suggests a role for CXCL8 in promoting nuclear translocation of viral DNA. Another possibility explaining the observed results may be a rise in number of infected cells following CXCL8 treatment. It may, thus, contribute to increase in 2-LTR circles as a whole instead of increased 2-LTR circle levels per cell. In contrast, studies have shown 2-LTR circles persisting in long-lived nondividing cells and in patients undergoing ART [44], [76]. In such cases, 2-LTR circles cannot be considered as a direct marker for ongoing viral replication. Since our present study focused on acute infection, analysis of 2-LTR circles is a reliable method to assess early steps of HIV-1 infection. Therefore, our results establish that CXCL8 treatment increases nuclear transport of viral cDNA and promotes viral infection.

Activation of NF-κB leads to expression of various chemokines including CXCL8 in astrocytes, macrophages and microglia [62], [77], [78]. Interestingly, the secreted chemokines may exhibit feedback activation of NF-κB to maintain their constitutive expression [79], [80]. Also, the NF-κB binding regions in HIV-1 LTR promoter are well documented and thus transcription factor NF-κB is implicated in regulating HIV-1 LTR activity [34]. Our findings explore the role of CXCL8 in NF-κB activation. We demonstrated that CXCL8 was efficient in activating signaling cascades leading to nuclear translocation of NF-κB in macrophages, even in the absence of HIV-1 infection. The next logical question was to investigate the role of translocated NF-κB in HIV-1 LTR promoter activity, for which we utilized both the U937 system and macrophages. We found that p65 subunit of NF-κB was important for CXCL8-induced HIV-1 LTR activity. Interestingly, NF-κB activation is linked to generation of inflammatory responses and release of CXCL8 in U937 and macrophages [81], [82]. Therefore, present data indicate that NF-κB induces CXCL8 expression that in turn further activates NF-κB, and this loop is beneficial for viral replication. More detailed investigation could be carried out to unravel molecular signaling involved upstream of NF-κB, following CXCL8 treatment in macrophages.

In our study, we observed significant differences in induction of HIV-1*p24* levels by HIV-1_ADA_ infection in comparison to HIV-1_JRFL_ infection in both MDM and microglia. A plausible explanation could be the variation in the source of two viral strains; HIV-1_ADA_ being blood-derived [40] and HIV-1_JRFL_ being brain-derived [83]. It has been proposed that HIV-1 compartmentalization in the brain makes the virus evolve independently, giving rise to differential tropism of brain-derived isolates [84], [85]. A potential biological explanation may be variation in NF-κB binding sites in the HIV-1 LTR promoter region of HIV-1_ADA_ and HIV-1_JRFL_, leading to differential replication and viral infectivity in microglia and MDM following CXCL8 treatment. However, there are no reported differences in NF-κB binding sites amongst viruses belonging to subtype B [86] including HIV-1_ADA_ and HIV-1_JRFL_. A recent report suggests additional NF-κB binding site in HIV-1 subtype C when compared to two canonical sites in HIV-1 subtype B [86]. Further studies are necessary to explain the observed results.

Since CXCL8 is a key mediator of inflammation during HIV-1 infection and our study highlights its importance in promoting HIV-1 replication, blocking the production or effects of CXCL8 can be a therapeutic strategy. Complex signaling pathways interact to produce CXCL8 in various cellular systems, therefore, blocking the production completely may not be feasible *in vivo*. Instead, indirect approaches need to be employed. A recent study demonstrated use of dominant-negative CXCL8 decoy proteins *in vivo* to block CXCL8-induced inflammatory cascade [87]. In another approach, the receptors CXCR1 and CXCR2 may be targeted to block the effects of CXCL8 in brain microenvironment. Blocking of CXCL8 receptor CXCR1 by inhibitor reparixin has been shown to reduce short-term neutrophil infiltration and infarct size. Inhibiting both CXCR1 and CXCR2 decreased polymorphonuclear lymphocyte infiltration and improved neurological function in permanent and transient rat cerebral ischemia [88], [89]. Reparixin has been tested in human clinical trials as a combinatorial therapy in diabetes and cancer [90]. Our findings compliment these studies and confirm the role of chemokine CXCL8 and receptors CXCR1/2 in regulating HIV-1 infection in MDM and microglia.

## Conclusion

In summary, our data suggest a mechanism where elevated CXCL8 levels during HIV-1 infection of the brain promote HIV-1 infection and HIV-1*p24* release in human macrophages and microglia. CXCL8 binds to receptors CXCR1/2 and initiates NF-κB translocation into the nucleus, thereby promoting HIV-1 LTR promoter activity, which in turn increases viral gene expression. Considering its affect on HIV-1 infection in macrophages and microglia, CXCL8 can be a potential therapeutic target for controlling persistence of viral infection in the brain. CXCR1/2 inhibition in combination with ART might prove to be a better therapy for control of neuroinflammation in HIV-1 infection.
